# The Function of the Chemokine Receptor CXCR6 in the T Cell Response of Mice against *Listeria monocytogenes*

**DOI:** 10.1371/journal.pone.0097701

**Published:** 2014-05-15

**Authors:** Kira Heesch, Friederike Raczkowski, Valéa Schumacher, Stefanie Hünemörder, Ulf Panzer, Hans-Willi Mittrücker

**Affiliations:** 1 Institute for Immunology, University Medical Center Hamburg-Eppendorf, Hamburg, Germany; 2 3rd Department of Medicine, University Medical Center Hamburg-Eppendorf, Hamburg, Germany; Cincinnati Children's Hospital, United States of America

## Abstract

The chemokine receptor CXCR6 is expressed on different T cell subsets and up-regulated following T cell activation. CXCR6 has been implicated in the localization of cells to the liver due to the constitutive expression of its ligand CXCL16 on liver sinusoidal endothelial cells. Here, we analyzed the role of CXCR6 in CD8^+^ T cell responses to infection of mice with *Listeria monocytogenes*. CD8^+^ T cells responding to listerial antigens acquired high expression levels of CXCR6. However, deficiency of mice in CXCR6 did not impair control of the *L. monocytogenes* infection. CXCR6-deficient mice were able to generate listeria-specific CD4^+^ and CD8^+^ T cell responses and showed accumulation of T cells in the infected liver. In transfer assays, we detected reduced accumulation of listeria-specific CXCR6-deficient CD8^+^ T cells in the liver at early time points post infection. Though, CXCR6 was dispensable at later time points of the CD8^+^ T cell response. When transferred CD8^+^ T cells were followed for extended time periods, we observed a decline in CXCR6-deficient CD8^+^ T cells. The manifestation of this cell loss depended on the tissue analyzed. In conclusion, our results demonstrate that CXCR6 is not required for the formation of a T cell response to *L. monocytogenes* and for the accumulation of T cells in the infected liver but CXCR6 appears to influence long-term survival and tissue distribution of activated cells.

## Introduction

*Listeria monocytogenes* is a Gram-positive, rod-shaped bacterium with ubiquitous distribution in nature. Infection mainly occurs by contaminated food. Risk groups include immunocompromised and old persons, pregnant women and neonates. Infection of mice with *L. monocytogenes* causes rapid activation of the innate immune system, which is essential for the restriction of bacterial replication. Due to its intracellular growth, *L. monocytogenes* induces a strong CD8^+^ T cell response. These CD8^+^ T cells accumulate in spleen and liver and are mainly responsible for bacterial clearance and for effective protection after reinfection [1], [2]. The mechanisms regulating CD8^+^ T cell accumulation in the infected liver are only partially understood [3]. Recruitment of T cells to sites of infection is controlled by the local expression of addressins, adhesion molecules and pro-inflammatory chemokines. On an mRNA level, activated CD8^+^ T cells in *L. monocytogenes* infection express relatively high levels of the chemokine receptors CCR2, CCR5, CXCR3 and CXCR6 which respond to pro-inflammatory chemokines (unpublished results). However, there are only few studies on the role of these chemokine receptors in *L. monocytogenes*-infection. CXCR3 was shown to control migration of CD8^+^ T cells within different compartments of the spleen and to regulate differentiation processes of these cells. However, absence of CXCR3 did not reduce liver accumulation of CD8^+^ T cells during acute infection [4]. CCR5-deficient mice showed impaired control of *L. monocytogenes* [5], though a second study could not confirm enhanced susceptibility and further showed that CCR5-deficient mice mounted normal listeria-specific CD8^+^ T cell responses [6].

The chemokine receptor CXCR6 (CD186), also known as Bonzo, STRL3 or TYMSTR, was originally described as a co-receptor for SIV and HIV [7]–[9]. It is expressed on subsets of CD4^+^ T cells, NK cells, NKT cells, and plasma cells [10]. CXCR6 is expressed at very low levels on naive CD8^+^ T cells [11], but it is up-regulated after activation [12]–[14]. The only known ligand for CXCR6 is CXCL16, also known as SR–PSOX (scavenger receptor that binds phosphatidyl serine and oxidized lipoprotein) [11], [15], [16]. Similar to CX_3_CL1/fractalkine [17], CXCL16 is expressed with a transmembrane domain and can function as a membrane-bound chemokine or adhesion molecule [11], [15]. Transmembrane CXCL16 is expressed by macrophages, monocytes, dendritic cells, B cells and sinusoidal endothelium cells of the liver [7], [18]. Under certain conditions, soluble CXCL16 is generated by protease mediated shedding of the membrane-bound form by ADAM-10 or ADAM-17 [19].

In this study, we investigated the role of CXCR6 in CD8^+^ T cell activation, differentiation, and migration during the immune response of mice against *L. monocytogenes*. Listeria-specific CD8^+^ T cells in spleen and liver acquired CXCR6 expression on their surface. However, CXCR6 deficiency did not impair control of *L. monocytogenes* infection and CXCR6-deficient mice generated normal CD4^+^ and CD8^+^ T cell responses and showed similar accumulation of these cells in the liver. In T cell transfer assays, early accumulation of activated listeria-specific CD8^+^ T cells in the liver depended on the expression of CXCR6. However, CXCR6 became dispensable and at the peak of response CXCR6-deficient and control CD8^+^ T cells accumulated to similar extend in the liver. When transferred CD8^+^ T cells were followed over extended time periods, CXCR6-deficiency resulted in altered tissue distribution and reduced persistence of CD8^+^ T cells indicating a function of CXCR6 in maintaining long-term survival of CD8^+^ T cells.

## Materials and Methods

### Mice

C57BL/6 mice (The Jackson Laboratory), CD90.1-congenic C57BL/6 mice (B6.PL-Thy1a/CyJ; The Jackson Laboratory), RAG1^−/−^ mice (The Jackson Laboratory), OT–I mice [20], and CXCR6^GFP/GFP^ mice [21] were bred under specific-pathogen-free conditions at the animal facility of the University Medical Center Hamburg-Eppendorf. Experiments were conducted according to the German animal protection law. Experiments were approved by the Behörde für Gesundheit und Verbraucherschutz of the City of Hamburg under the permits 56/12 and 99/10. Animals were housed in individually ventilated cages under 12 h light/dark cycles and constant temperature. Water and food was provided ad libitum. During acute infection, mice were controlled daily. Animals with overt symptoms of disease were euthanatized to avoid suffering. Animals were euthanatized with CO_2_.

### Infection of mice with *L. monocytogenes*

Age (>8 weeks) and sex-matched mice were infected intravenously (i.v.) or intraperitoneally (i.p.) with indicated doses of the wild type *L. monocytogenes* strain EGD (*Lm*) or of a *L. monocytogenes* strain expressing ovalbumin (*Lm*OVA) [22]. Bacterial inocula were controlled by plating serial dilutions on tryptic soy broth (TSB) agar plates.

### Isolation of cells

Lymphocytes from spleen and lymph nodes were obtained by mashing the organs through a cell sieve into PBS followed by erythrocyte lysis with lysing buffer (155 mM NH_4_Cl, 10 mM KHCO_3_, 100 µM EDTA [pH 7.2]). Liver and lung were perfused with PBS. For purification of lymphocytes from the liver, the liver cell suspension was additionally separated using a 40%/70% Percoll gradient (Biochrom AG, Berlin, Germany) before erythrocyte lysis. Lungs were cut into small pieces and digested with 2 mg/ml collagenase D (Roche, Penzberg, Germany) and 100 µg/ml DNAse I (Roche) in complete RPMI1640 medium (RPMI1640 medium supplemented with 10% heat-inactivated fetal calf serum, 2 mM glutamine, 50 µM β-mercaptoethanol, and 50 mg/ml gentamycin) before lysing red blood cells. Lymphocytes from bone marrow were isolated out of one femur and erythrocytes were lysed.

### Determination of listeria titers

Bacterial burdens in spleen and liver were determined by homogenization of organs in 0.1% Triton X–100. Serial dilutions of homogenates were plated on TSB agar plates and colonies were counted after 24 h of incubation at 37°C.

### *In vitro* stimulation of T cells

For the determination of cytokine production, 2×10^6^ lymphocytes were incubated with ovalbumin peptide (OVA_257-264_, SIINFEKL; JPT Peptide Technologies GmbH, Berlin) for specific stimulation of CD8^+^ T cells and with listeriolysin O peptide (LLO_189-201_, NEKYAQAYPNVS; JPT Peptide Technologies GmbH, Berlin) for specific stimulation of CD4^+^ T cells in complete RPMI1640 medium for 4 h at 37°C. To prevent protein secretion, 10 µg/ml Brefeldin A (BFA; Sigma) was added for the final 3.5 h of culture.

For the analysis of proliferation, 4×10^5^ cells from spleen were incubated for 3 d at 37°C with increasing concentrations of CXCL16 (3–300 ng/ml), with increasing concentrations of IL–15 (3–300 ng/ml) or with 2 µg/ml anti–CD3 mAb and 2 µg/ml anti–CD28 mAb.

### Flow cytometry

For extracellular cell-staining, lymphocytes were incubated with 10 µg/ml anti–CD16/CD32 mAb (anti–FcγRII/III; BioXCell, West Lebanon) and 1∶100 normal rat serum (NRS; Jackson Laboratories, Bar Harbor) to minimize unspecific antibody binding and then stained with specific fluorochrome-conjugated mAbs for 20 min at 4°C. MAbs to CD8α (53–6.7), CD4 (RM4–5), CD90.1 (HIS51), CD90.2 (53–2.1), CD44 (IM7), CD62L (MEL–14), KLRG1 (2F1), IFN–γ (XMG1.2), TNF–α (MP6–XT22, TN3–19), PD–1 (J43), LAG3 (eBioC9B7W) and CD244 (2B4) were purchased from BioLegend (San Diego), eBioscience (San Diego), or BD (Heidelberg). Cells were measured with a FACS CantoII flow cytometer (BD, Heidelberg). Results were analyzed with the DIVA Software (BD, Heidelberg) and the FlowJo Software (Tree Star, Ashland). Debris, doublets, and dead cells were excluded from analysis. Dead cells were identified by staining with Dapi (4′,6-diamidino-2-phenylindole; Merck, Darmstadt) or AlexaFluor–750 succinimidyl ester (AF750; Invitrogen, Karlsruhe).

### Intracellular staining

For intracellular cytokine staining, cells were fixed with 2% paraformaldehyde in PBS for 15 min at room temperature and washed with 0.2% BSA (bovine serum albumin) in PBS. Cells were blocked with anti–CD16/32 mAb and NRS in PBS containing 0.1% BSA and 0.3% saponin (Sigma) and stained with fluorochrome-conjugated mAb in the same buffer.

### Flow cytometry-based assays

*In vivo* proliferation was determined by BrdU (Bromodeoxyuridine; BD, Heidelberg) incorporation. Mice received 1 mg BrdU i.p. 1 d before analysis. Anti–BrdU mAb was used according to the manufacturer's protocol. *In vitro* proliferation was measured by staining of cells with the proliferation dye eFluor 670 (eF670; eBioscience, San Diego). 5×10^7^ cells were incubated in PBS containing 5 µM eF670 and 0.2% BSA for 15 min at 37°C. Apoptosis was determined by using the FLICA Kit (Immunochemistry Technologies, Bloomington) according to the manufacturer's protocol.

### Competitive T cell transfer experiments

CD8^+^ T cells from spleens of CXCR6^GFP/GFP^ OT–I mice and CD90 congenic wt OT–I mice were purified by negative magnetic activated cell sorting (MACS; Miltenyi Biotech, Bergisch Gladbach) according to the manufacturer's protocol. Wt OT–I (CD90.1^+^ CD90.2^+^) and CXCR6^GFP/GFP^ OT–I (CD90.1^−^ CD90.2^+^) CD8^+^ cells were mixed in a 1∶1 ratio and a total of 5×10^4^ cells were injected i.v. into congenic wt (CD90.1^+^ CD90.2^−^) or RAG1^−/−^ mice. Recipient mice were infected with 1×10^5^ *Lm*OVA i.p. 4 h before transfer.

### Statistical analysis

All statistical analysis was performed with GraphPad Prism software (La Jolla). Differences between two groups of mice were analyzed by Mann-Whitney *U* test (bacterial burden) or student t-test. Differences between three or more groups were analyzed by one-way analysis of variance (ANOVA) with Dunnett post-test. A p-value of<0.05 was considered significant (*, p<0.05; **, p<0.01; ***, p<0.001).

## Results

### CXCR6 expression on CD8^+^ T cells during *L. monocytogenes* infection

**Figure 1:**
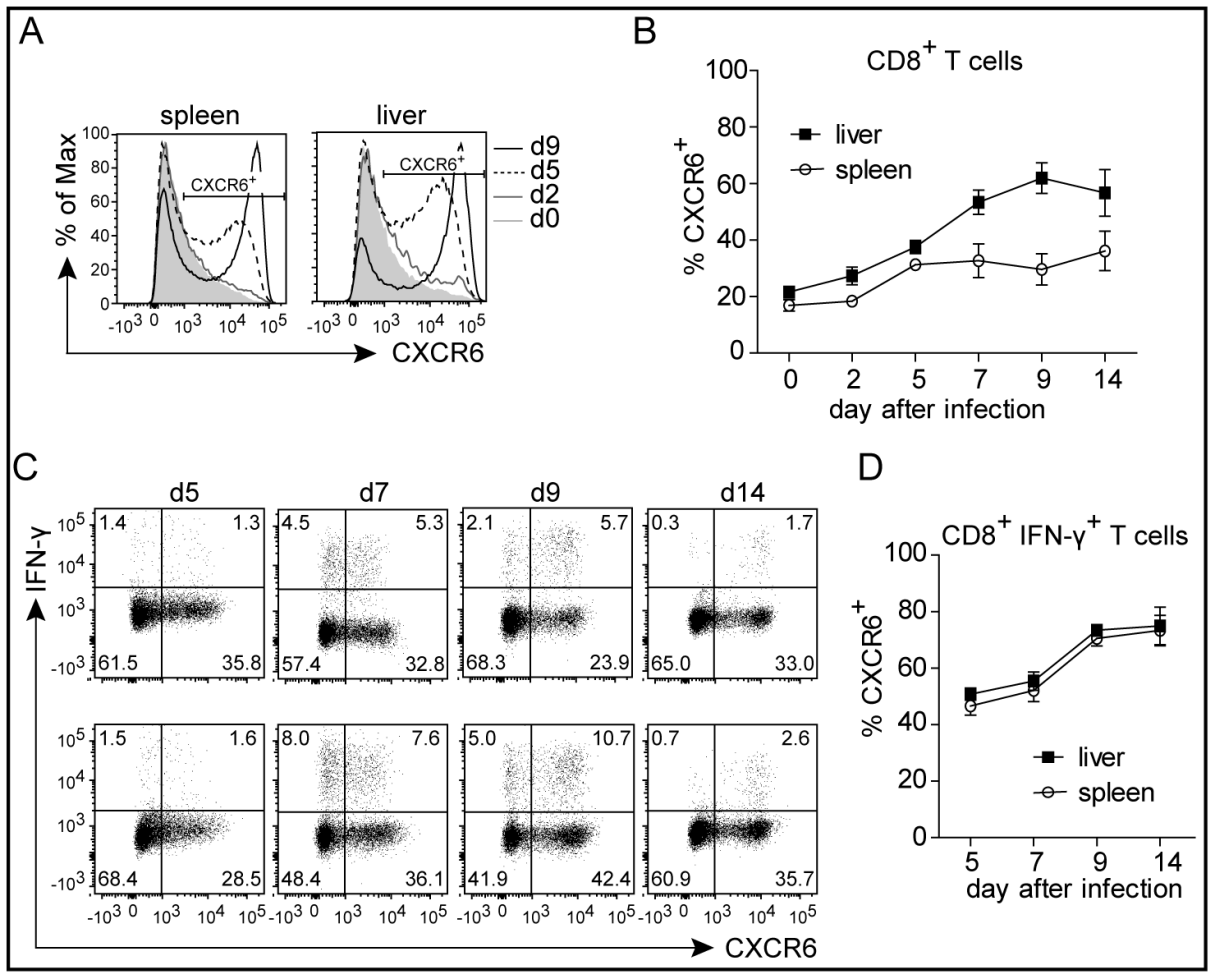
CXCR6 expression on CD8^+^ T cells during *L. monocytogenes* infection. CXCR6^+/GFP^ mice were infected with 1×10^4^ *Lm*OVA i.v. and cells from spleens and livers were analyzed at indicated time points. (A) Representative histogram of CXCR6 expression by CD8^+^ T cells. (B) Expression of CXCR6 by CD8^+^ T cells. (C) Representative dot plots of CXCR6 and IFN–γ expression by CD8^+^ T cells. Upper row: spleen, lower row: liver (D) Expression of CXCR6 by IFN–γ^+^ listeria-specific CD8^+^ T cells. Symbols in (B) and (D) give the mean ± SEM, n≥5 and are representative for two experiments.

Expression of the chemokine receptor CXCR6 has been described for activated CD8^+^ T cells [12]–[14].We therefore tested whether the infection of mice with *L. monocytogenes* causes changes in the expression level of CXCR6 on CD8^+^ T cells. For the determination of CXCR6 expression, we used heterozygous GFP knock-in mice, in which one allele of the *CXCR6* gene has been replaced by enhanced GFP [21]. In naive mice, we observed CXCR6 expression on approx. 20% of CD8^+^ T cells in spleen and liver. After infection of mice with *L. monocytogenes*, CD8^+^ T cells from spleen and liver showed up-regulation of CXCR6 (Fig. 1A, 1B). Up-regulation of CXCR6 was particular prominent on CD8^+^ T cells with an activated CD44^+^CD62L^−^ phenotype (Fig. S1). In the liver, even in the non-infected state, most of CD44^+^CD62L^−^ CD8^+^ T cells were CXCR6^+^. Mice were infected with a *L. monocytogenes* strain recombinant for ovalbumin (*Lm*OVA). Short term stimulation with the immunodominant peptide OVA_257-264_ enabled detection of CD8^+^ T cells generated in response to infection via their expression of IFN–γ (Fig. 1C, 1D). At d5, the first time point of detection of specific CD8^+^ T cells, 50% of IFN–γ^+^ cells co-expressed CXCR6. CXCR6 reached a high expression level on IFN–γ^+^ CD8^+^ T cells at the peak of the CD8^+^ T cell response at day 9 and remained at this high level at d14, when the specific CD8^+^ T cell population had started to contract. Thus, compared to IFN–γ expression, CXCR6 expression on CD8^+^ T cells was delayed.

### CXCR6 is not essential for a protective immune response against *L. monocytogenes*

**Figure 2:**
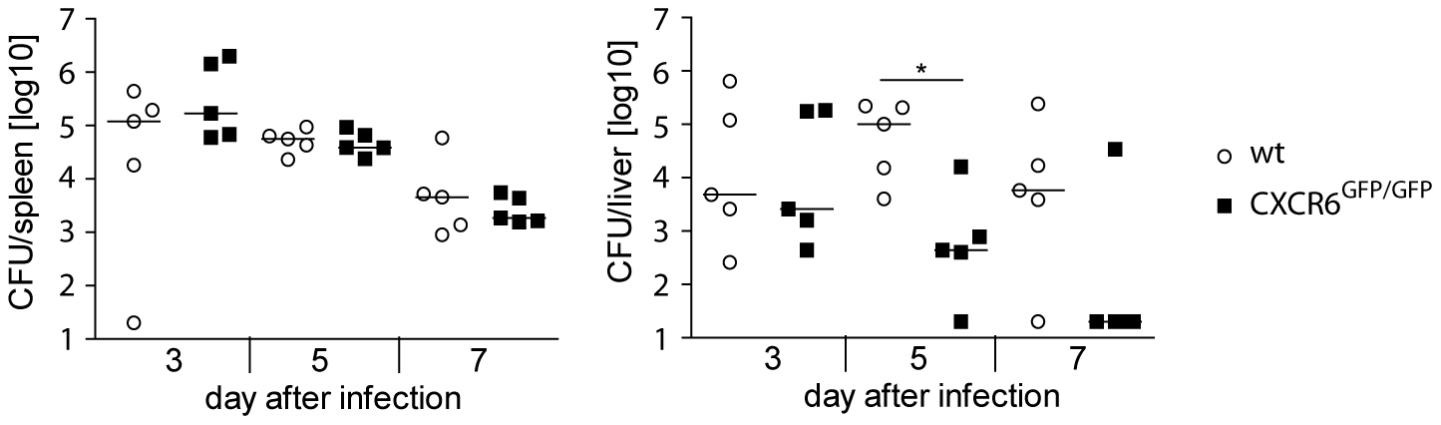
Control of *L. monocytogenes* in CXCR6-deficient mice. Wt and CXCR6^GFP/GFP^ mice were infected with 1×10^4^ *Lm* i.v. and listeria titers in spleens and livers were determined at indicated time points. Colony forming units (CFU) for individual mice and the median of one representative experiment of two are shown, n = 5. Detection limit was 20 CFU. *, p<0.05.

Control of *L. monocytogenes* depends on innate immune mechanisms as well as on effective CD4^+^ and CD8^+^ T cell responses. To investigate the role of CXCR6 in the control of infection, CXCR6-deficient (CXCR6^GFP/GFP^) and wt mice were infected with *L. monocytogenes* and the bacterial burdens in spleen and liver were determined (Fig. 2). In the spleen, CXCR6^GFP/GFP^ and wt mice had similar bacterial titers at all analyzed time points. Interestingly, CXCR6^GFP/GFP^ mice showed lower listeria titers in the liver at d5 and d7 post infection. Slightly better protection was also observed in the liver of CXCR6^GFP/GFP^ mice after infection with a higher bacterial dose, although differences did not reach a level of significance (Fig. S2). Thus, control of listeria-infection occurred independent of CXCR6 and in the liver, absence of CXCR6 even improved clearance of bacteria.

**Figure 3:**
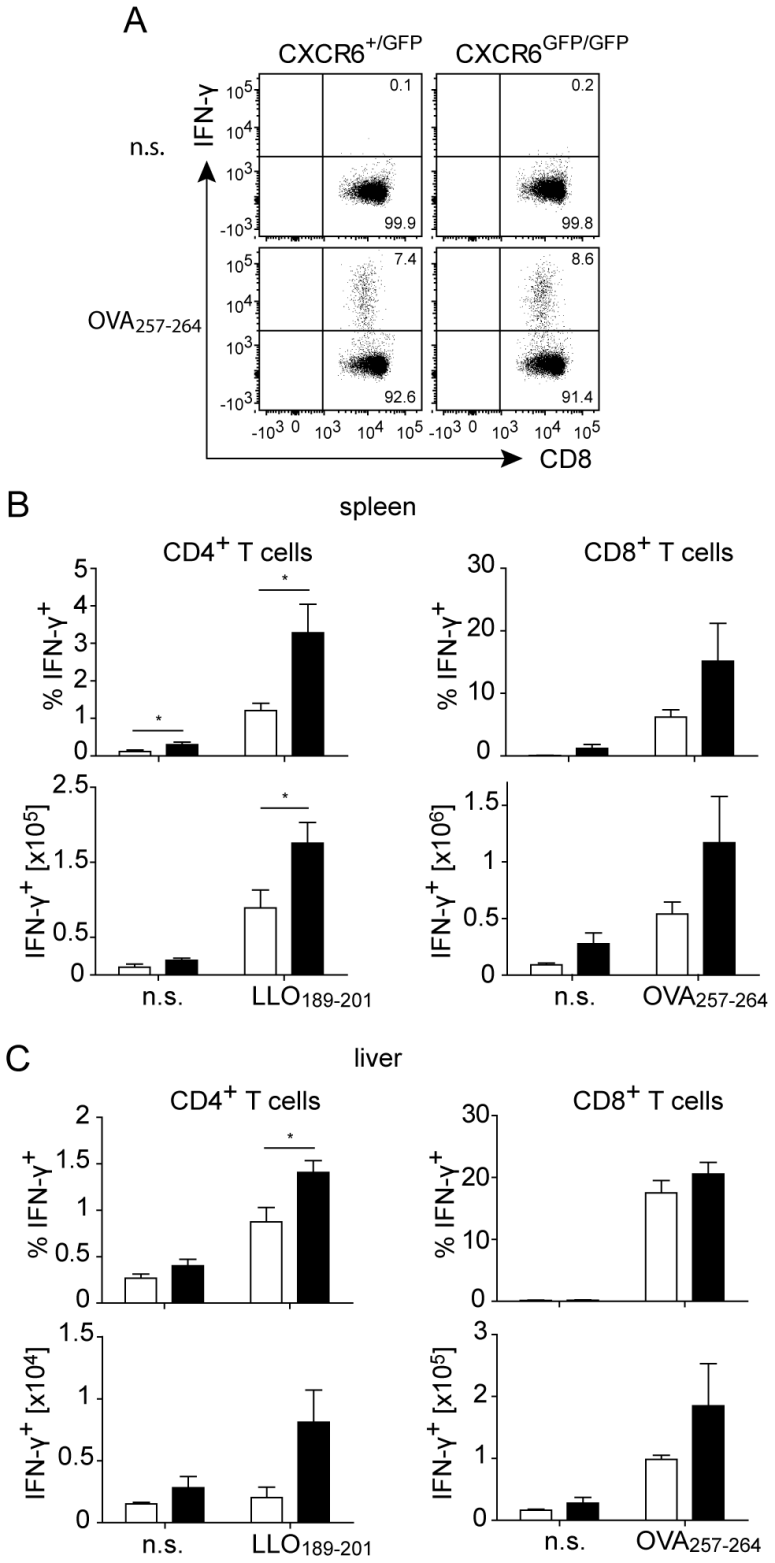
T cell responses of CXCR6-deficient mice against *L. monocytogenes.* CXCR6^+/GFP^ and CXCR6^GFP/GFP^ mice were infected with 1×10^4^ *Lm*OVA i.v. and spleens and livers were analyzed eight days p.i. Cytokine production was measured after stimulation of cells with LLO_189-201_ (CD4^+^ T cells) and OVA_257-265_ (CD8^+^ T cells). (A) Representative dot plots of IFN–γ expression in CD8^+^ T cells from spleen. Numbers show percentages of positive cells. (B, C) Percentages and numbers of IFN–γ^+^ CD4^+^ and IFN–γ^+^ CD8^+^ T cells in spleens and livers of infected CXCR6^+/GFP^ (white bars) and CXCR6^GFP/GFP^ (black bars) mice. Bars give mean ± SEM, n≥5. The experiment was repeated twice with consistent results. n.s., not stimulated; *, p<0.05.

To determine the role of CXCR6 in the induction of CD4^+^ and CD8^+^ T cell responses, we infected CXCR6^GFP/GFP^ and control (CXCR6^+/GFP^) mice with *Lm*OVA. On day 8, cells from spleen and liver were stimulated *in vitro* with the immunodominant peptides LLO_189-201_ (CD4^+^ T cells) and OVA_257-264_ (CD8^+^ T cells), and frequencies of IFN–γ^+^ cells were determined. We detected enhanced CD4^+^ T cell responses in spleen and liver of CXCR6^GFP/GFP^ mice when compared to control mice (Fig. 3A–C). CD8^+^ T cell responses in spleen and liver tended to be higher in CXCR6^GFP/GFP^ mice as well, however, differences did not reach a significant level. Overall, CXCR6-deficient mice were not impaired in the generation of CD4^+^ and CD8^+^ T cell responses to *L. monocytogenes*.

### CXCR6 controls early accumulation of activated CD8^+^ T cells in the liver

**Figure 4:**
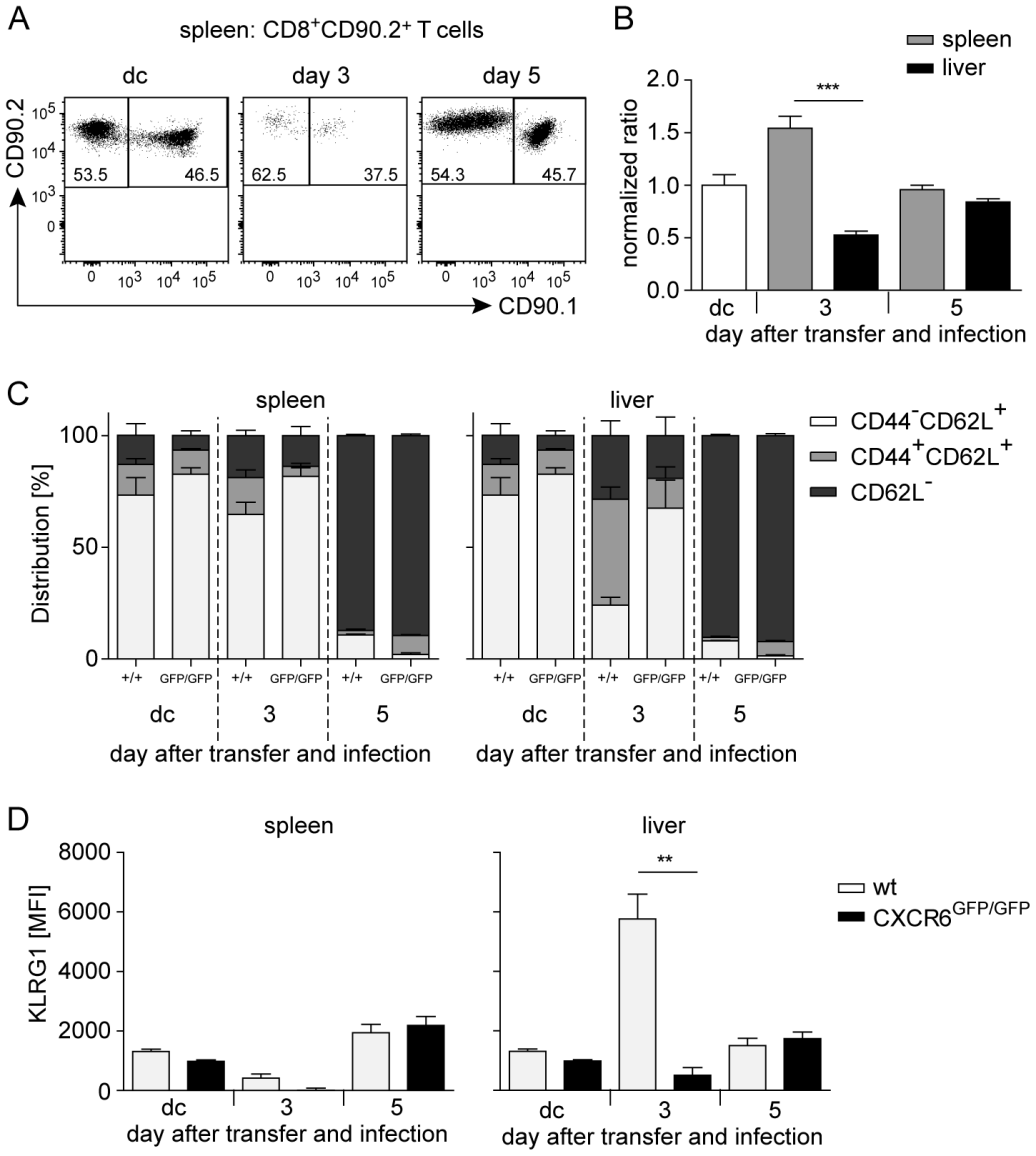
CXCR6 controls early accumulation of activated CD8^+^ T cells in the liver. Purified CD8^+^ T cells from wt OT–I and CXCR6^GFP/GFP^ OT–I mice were mixed in a ratio of 1∶1 and a total of 5×10^4^ cells were injected into congenic wt mice i.v. infected with 1×10^5^ *Lm*OVA. Transferred cells from spleens and livers of recipient mice were analyzed at day 3 and day 5 p.i. (A) Representative CD90.1 and CD90.2 staining of purified and mixed OT–I cells before (dc, donor cells) and three and five days after transfer. Numbers show percentages of positive cells. (B) Normalized ratio of transferred OT–I cells in spleens and livers of recipient mice on indicated time points. (C, D) Activation status of transferred OT–I cells. (C) Bars show distribution of CD44^−^CD62L^+^, CD44^+^CD62L^+^, and CD62L^−^ cells within wt and CXCR6^GFP/GFP^ OT–I cells. (D) Mean fluorescence intensity (MFI) of KLRG1 staining on transferred OT–I cells. Bars give mean ± SEM, n≥3. The experiment was repeated twice with consistent results. **, p<0.01; ***, p<0.001.

To restrict the analysis to the function of CXCR6 on CD8^+^ T cells, competitive T cell transfer assays were conducted. We transferred small numbers of an 1∶1 mixture of wt and CXCR6^GFP/GFP^ OVA-specific OT–I CD8^+^ T cells into *Lm*OVA-infected wt recipients. Differential expression for CD90.1 and CD90.2 allowed the distinction of wt OT–I cells (CD90.1^+^CD90.2^+^), CXCR6^GFP/GFP^ OT–I cells (CD90.1^−^CD90.2^+^) and recipient CD8^+^ T cells (CD90.1^+^CD90.2^−^) (Fig. 4A). At day 3 after transfer and infection, we found different percentages of wt and CXCR6^GFP/GFP^ OT–I cells in spleen and liver of recipient mice. Whereas the portion of CXCR6^GFP/GFP^ OT–I cells was higher in the spleen, the portion of wt OT–I cells was higher in the liver. The normalized ratio of transferred CXCR6^GFP/GFP^ to wt OT–I cells between spleen and liver was significantly different (Fig. 4B and Fig. S3A). Characterization of transferred CD8^+^ T cells revealed that the majority of wt OT–I cells in the liver displayed an activated CD44^+^CD62L^+^ or CD44^+^CD62L^−^ phenotype with enhanced KLRG1 expression (Fig. 4C, 4D and Fig. S3B, S3C). In contrast, CXCR6^GFP/GFP^ OT–I cells were largely CDD44^−^CD62L^+^. However, five days after transfer the populations of wt and CXCR6^GFP/GFP^ OT–I cells had equalized in spleen and liver of recipient mice and both T cell populations displayed a similar phenotype. These results demonstrate that early after infection with *L. monocytogenes*, CXCR6 was important for migration of activated CD8^+^ T cells to the liver but CXCR6 became dispensable at later time-points of infection.

### CXCR6 controls tissue distribution and survival of pre-activated CD8^+^ T cells

**Figure 5:**
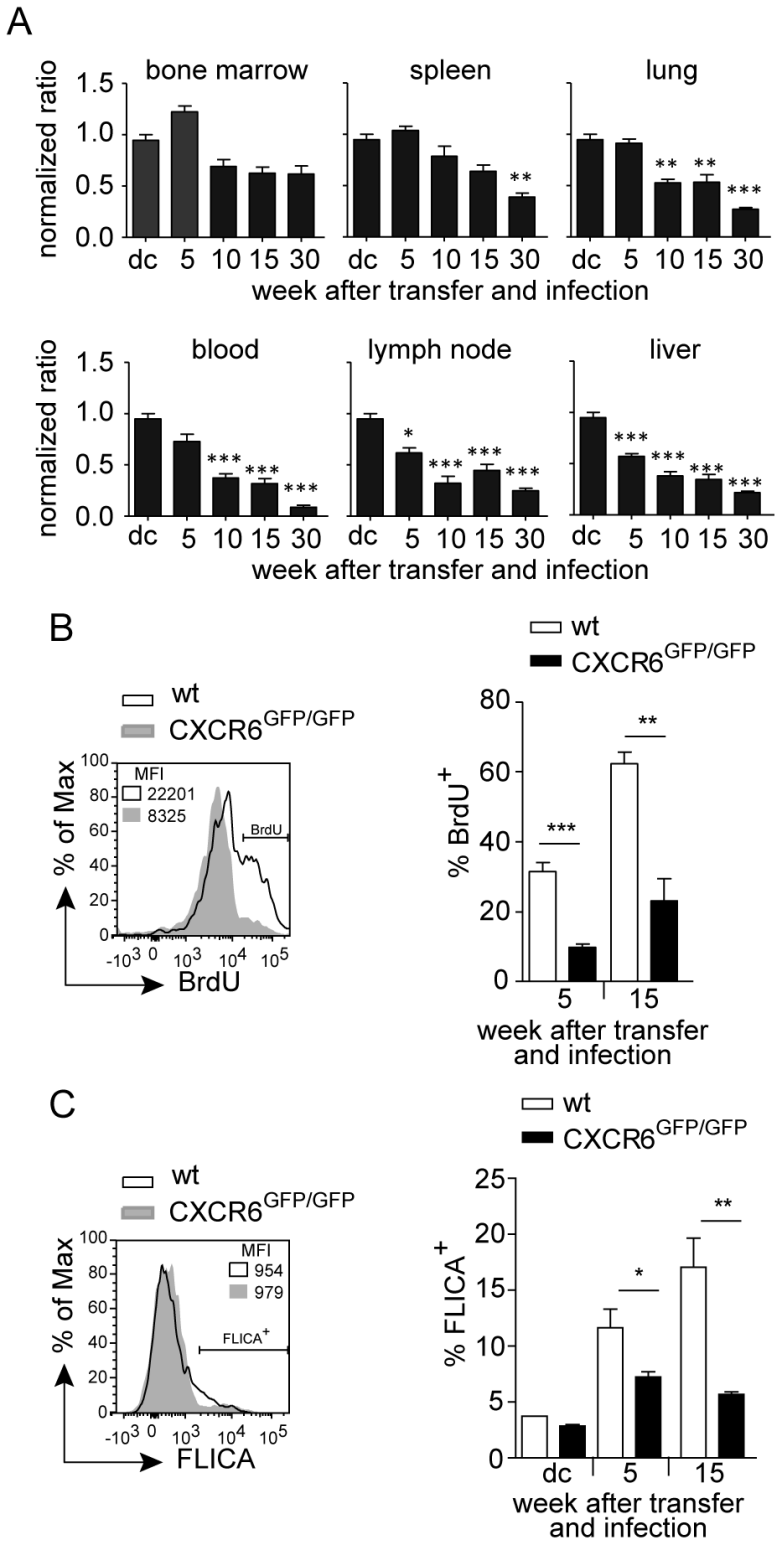
CXCR6 controls tissue distribution and survival of pre-activated CD8^+^ T cells. Purified CD8^+^ T cells from wt OT–I and CXCR6^GFP/GFP^ OT–I mice were mixed in a ratio of 1∶1 and a total of 5×10^4^ cells were injected into RAG1^−/−^ mice i.v. infected with 1×10^5^ *Lm*OVA. Transferred cells of recipient mice were analyzed at 5, 10, 15 and 30 weeks p.i. (A) Normalized ratios of transferred OT–I cells in bone marrow, spleens, lungs, blood, lymph nodes and livers of recipient mice on indicated time points. (B) BrdU incorporation by transferred cells. Mice received BrdU one day before analysis. Representative histogram for OT–I cells 5 weeks after transfer and combined results for 5 and 15 weeks after transfer. (C) FLICA binding by transferred cells. Representative histogram for OT–I cells 5 weeks after transfer and combined results for donor cells (dc) before transfer and at 5 and 15 weeks after transfer. Bars give mean ± SEM of combined results from two independent experiments, n≥6. MFI, mean fluorescence intensity; *, p<0.05; **, p<0.01; ***, p<0.001.

CXCR6 has been shown to be required for the long-term persistence of memory NK cells [23]. To determine the role of CXCR6 for CD8^+^ T cell persistence and tissue distribution over extend time periods, we used the competitive T cell transfer assay. Although CXCR6^GFP/GFP^ mice were backcrossed to the C57BL/6 background, we cannot fully exclude rejection of transferred T cells in wt recipients, particularly when transferred cells express high GFP levels [24]. Therefore, competitive transfers were conducted with RAG1^−/−^ mice as recipients (Fig. 5A and Fig. S4A). Five weeks after transfer and *Lm*OVA infection, we observed similar frequencies of wt and CXCR6^GFP/GFP^ OT–I cells in spleen, blood, lung and bone marrow. Compared to wt OT–I cells, CXCR6^GFP/GFP^ OT–I cells remained relatively stable in bone marrow and spleen of recipient mice. In contrast, the proportion of CXCR6^GFP/GFP^ OT–I cells steadily declined in blood, lymph node, lung and liver, and 30 weeks after transfer, wt OT–I cells largely outnumbered CXCR6^GFP/GFP^ OT–I cells in these tissues.

Transferred T cells were analyzed at different time point for their activation status. For all time points analyzed, we observed similar expression profiles of CD44 and CD62L on wt and CXCR6^GFP/GFP^ OT–I T cells, and following peptide stimulation both cell populations showed comparable expression of IFN–γ and TNF–α (Fig. S4B–E). The rate of proliferation of transferred T cells was measured by determination of BrdU incorporation (Fig. 5B). Five and 15 weeks after co-transfer and infection, we observed significantly reduced BrdU incorporation in CXCR6^GFP/GFP^ OT–I T cells. In parallel, apoptosis of cells was analyzed with FLICA (fluorescent labeled inhibitor of caspases), which irreversibly binds activated caspases. With this assay, CXCR6^GFP/GFP^ T cells showed reduced apoptosis when compared to wt T cells (Fig. 5C). These results indicate that transferred wt OT–I T cells and CXCR6^GFP/GFP^ OT–I T cells differ in their turnover, with increased proliferation but also enhanced apoptosis in wt cells. Transferred T cells were also characterized for the expression of the exhaustion markers PD–1, LAG3 and CD244 (Fig. S5A, S5B). Expression of PD–1 and CD244 were largely similar on wt and CXC6^GFP/GFP^ OT–I cells, although we observed some variability at different time points, and LAG3 was consistently lower on CXCR6^GFP/GFP^ cells. Thus there was no indication for increased exhaustion of transferred CXCR6^GFP/GFP^ cells.

Finally, we tested whether the CXCR6 ligand CXCL16 could directly induce proliferation and survival in CD8^+^ T cells. Purified CD8^+^ T cells were eF670 labeled and incubated with different concentrations of CXCL16 or as controls with IL–15, anti–CD3 mAb plus anti–CD28 mAb or a combination of CXCL16 and IL–15 (Fig. S6A). IL–15 caused a dose dependent proliferation as determined by the loss of eF670 staining. In contrast, CXCL16 failed to cause T cell proliferation and did not enhance the proliferation induced by IL–15. To analyze the role of CXCL16 on survival of cells, spleen cells from wt and CXCR6^GFP/GFP^ mice were mixed with a CD8^+^ T cell ratio of 1∶1 and incubated for three days (Fig. S6B). Characterization of cells revealed a significant reduction of CXCR6^GFP/GFP^ CD8^+^ T cells compared to wt CD8^+^ T cells. The addition of CXCL16 did not further change the ratio of wt to CXCR6^GFP/GFP^ CD8^+^ T cells. An explanation could be that spleen cells already provided saturating amount of surface bound or soluble CXCL16. Even after stimulation of proliferation with anti–CD3 plus anti–CD28 mAbs, there was still some disadvantage of CXCR6^GFP/GFP^ CD8^+^ T cells. In summary, the interaction of CXCL16 with CXCR6 did not induce proliferation in CD8^+^ T cells but could maintain survival in these cells.

### CXCR6 is not essential for a protective secondary response against *L. monocytogenes*

**Figure 6:**
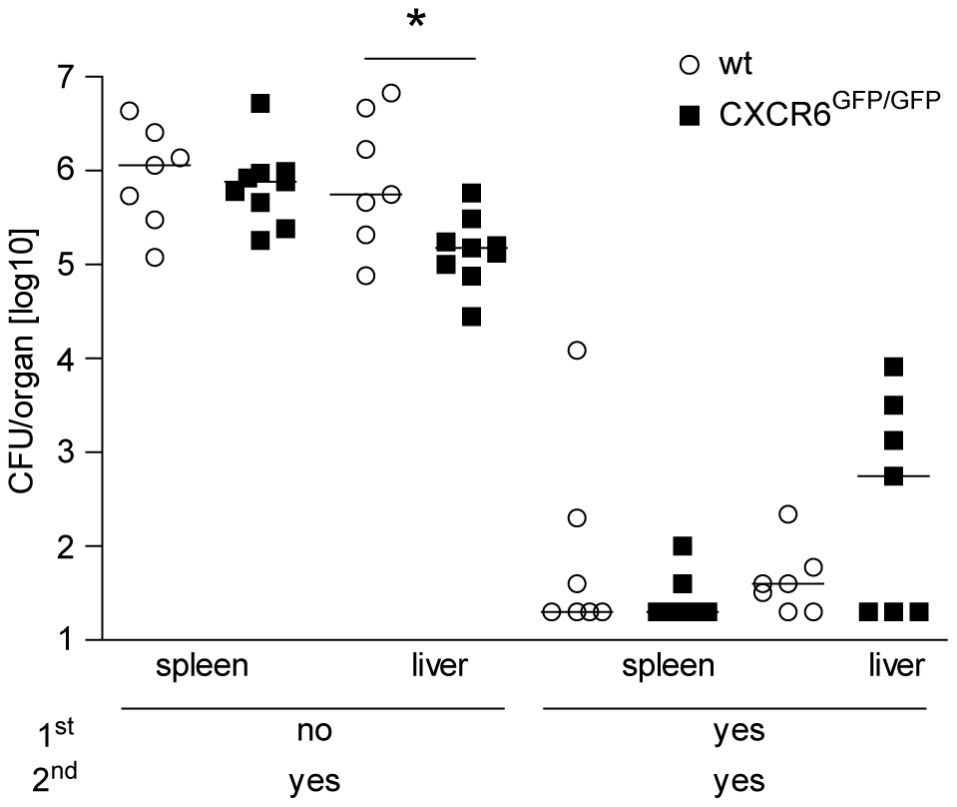
Control of secondary *L. monocytogenes* infection in CXCR6-deficient mice. Wt and CXCR6^GFP/GFP^ mice were primary infected with 5×10^3^ *Lm* i.v. and 60 days later challenged with 1×10^5^ *Lm* i.v. Two days after secondary infection, listeria titers in spleen and liver were determined. Colony forming units (CFU) for individual mice and the median of one representative experiment of two are shown, n≥7. Detection limit was 20 CFU. *, p<0.05.

Acquired protection against secondary *L. monocytogenes* infection mainly depends on specific CD8^+^ T cells generated during primary infection. To test whether the alterations in the maintenance of CD8^+^ T cells impair the control of *L. monocytogenes* following secondary infection, wt and CXCR6^GFP/GFP^ mice were challenged 60 days after primary infection with a high dose of *L. monocytogenes* (Fig. 6). Compared to naive mice, both control and CXCR6^GFP/GFP^ mice showed profoundly reduced listerial titers in spleen and liver two days post challenge infection. Although we observed slightly higher titers in the liver of secondary infected CXRC6^GFP/GFP^ mice, the difference did not reach the level of significance.

## Discussion

A population of CXCR6^+^ CD8^+^ T cells was detected in naive mice and mainly confined to CD8^+^ cells with a pre-activated CD44^+^CD62L^−^ phenotype. In the liver, the majority of CD44^+^CD62L^−^ CD8^+^ T cells was CXCR6^+^, indicating that under homeostatic conditions, CXCR6^+^ CD8^+^ T cells preferentially accumulate in the liver, probably due to the constitutive expression of CXCL16 on liver sinusoidal endothelium cells (LSEC) [11], [25]. Following *L. monocytogenes* infection, CXCR6 was up-regulated on activated CD8^+^ T cells and the majority of CD8^+^ T cells responding to listerial antigens acquired a CXCR6^+^ phenotype. Interestingly, CXCR6 expression was delayed when compared to the expression of IFN–γ, which would indicate that expression of CXCR6 and IFN–γ are independently regulated in CD8^+^ T cells. However, it has to be considered that although GFP expression is under the control of the *Cxcr6* promoter, GFP and CXCR6 could still be differently regulated on mRNA and protein level. Thus, GFP might not fully reflect surface expression of the CXCR6 protein.

Surprisingly, CXCR6-deficient mice showed better control of *L. monocytogenes* infection in the liver and equal or even stronger CD4^+^ and CD8^+^ T cell responses in spleen and liver. The reason for this improved response is currently unclear. We consider it unlikely that the CXCL16/CXCR6 interaction is directly suppressive to cells involved in the control of *L. monocytogenes*. However, CXCR6-deficient mice could either lack suppressive cells or show a compensatory response to the absence of a protective cell population, which might then even exceed the response observed in wt mice. CXCR6-deficient mice show profoundly reduced numbers of NKT cells in the liver [26] and might also lack certain NK cell subsets in this organ [23]. NKT cells are regarded as pro-inflammatory cells and as protective in bacterial infections [27]. However, in *L. monocytogenes* infection the role of NKT cells is unclear with studies showing better as well as diminished control of listeria in the absence of NKT cells [28]–[30]. In addition, there is also evidence for a suppressive role of NKT cells [31]–[33] and the CXCL16/CXCR6 axis might be a central regulatory element of this function. We could recently demonstrate that absence of CXCR6 causes aggravation of autoimmune kidney inflammation which was mainly due to diminished recruitment of regulatory NKT cells to the kidney [34]. Thus, reduced numbers of NKT cells in CXCR6-deficient mice could be responsible for the altered response to *L. monocytogenes*. For a hepatic NK cell population, it was proposed that CXCR6 is required to locate these cells to a suppressive environment (e.g. in the close proximity to LSEC) and thereby restrict their reactivity [23]. If a similar mechanism suppresses T cell activity, absence of CXCR6 might result in enhanced T cell responses in the liver.

CXCR6-deficent mice showed equal or even slightly enhanced accumulation of listeria-specific CD4^+^ and CD8^+^ T cells in the liver, when compared to wt mice. T cell transfer assays confirmed this result for CD8^+^ T cells. Thus CXCR6 was not necessary for the massive recruitment of T cells to the infected liver. In this respect, our result from the *L. monocytogenes* infection differs from studies in a graft-versus-host disease model, in which absences of CXRC6 causes reduced accumulation of CD8^+^ T cells in the inflamed liver [14]. Activated CD8^+^ T cells express an array of chemokine receptors for pro-inflammatory chemokines such as CCR5 or CXCR3 [21], [35]–[37], and these receptors could compensate for the absence of CXCR6, particularly in the context of strong hepatic inflammation during *L. monocytogenes* infection. On the other hand, the liver is easily accessible even for naive T cells [38] and up-regulation of integrin ligands and addressins could be sufficient for recruitment of activated T cells even in the absence of chemokine receptor signaling. Such a scenario was recently proposed for the recruitment of inflammatory monocytes to the *L. monocytogenes*-infected liver [39]. Interestingly, we observed impaired hepatic accumulation of CXCR6-deficient CD8^+^ T cells at early time points after transfer and infection which mainly affected CD8^+^ T cells with a highly activated CD44^+^CD62L^−^KLRG1^+^ phenotype. Thus, CXCR6 appears to play a role in the early phase of infection but is subsequently replaced by other chemokine receptors or chemokine independent mechanisms of T cell recruitment up-regulated with ongoing infection.

Our long-term transfer studies revealed that CXCR6-deficient CD8^+^ T cells were outnumbered over time by wt CD8^+^ T cells. However, stability of the CXCR6-deficient cells differed in the analyzed tissues. The proportion of CXCR6-deficient CD8^+^ T cells had significantly decline by 10 weeks in blood, peripheral lymph nodes, lung and liver but remained relatively stable for several additional weeks in spleen and bone marrow. We did not observe significant changes in the activation status of wt and CXCR6-deficient CD8^+^ T cells as well as in their cytokine response to *in vitro* stimulation. In addition, compared to wt cells CXCR6-deficient CD8^+^ T cells expressed similar or even lower levels of surface markers associated with CD8^+^ T cell exhaustion. A role of CXCR6 in the maintenance of a cell population has been described for hepatic NKT cells and subsets of NK cells [23], [26]. Similar to our observation for CD8^+^ T cells, these studies also show that CXCR6 is necessary for maintaining the NK or NKT cell populations but does not affect the function of the cells.

Measuring the rates of proliferation and apoptosis of transferred CD8^+^ T cells revealed that wt cells showed both higher proliferation and enhanced apoptosis in comparison to CXCR6-deficient cells. Thus, enhanced stability of wt cells was associated with a higher turn-over rate of these cells. *In vitro*, CXCL16 was not able to induce proliferation in CD8^+^ T cells. However, CXCR6-deficient CD8^+^ T cells showed reduced survival when compared to wt cells. The addition of CXCLl6 did not further enhance survival of wt cells, however, it is well possible that cells present in the culture, such as DCs, already provide saturating amounts of the chemokine [11], [26]. CXCR6 might also provide survival signals for CD8^+^ T cells *in vivo*. It is also possible that CXCR6 does not directly induce survival but directs cells to sites where these signals are available. Such a function of CXCR6 was proposed for the maintenance of NK cell subsets in the liver [23]. In a similar way, CXCR3 can direct activated CD8^+^ T cells to areas in the spleen where further differentiation signals are provided [4]. Such an indirect mechanism might also explain why CXCR6-deficient CD8^+^ T cells show different stability in different tissues.

Despite alterations in tissue distribution and long-term stability of CXCR6-deficient CD8^+^ T cells in the competitive T cell transfer assay, CXCR6-deficient mice were not significantly impaired in the control of secondary *L. monocytogenes* infection. Thus, despite the alterations, the CD8^+^ memory T cell compartment was still sufficient for substantial protection, at least at day 60 post primary infection. Protection assays were conducted in wt and CXRC6-deficient mice and not in the transfer model. Since CXCR6-deficient mice mount stronger primary CD8^+^ T cell responses, it is possible that they also generate a larger memory cell population, which might compensate for impaired survival of memory CD8^+^ T cells. In agreement with this hypothesis, we observed similar frequencies of *L. monocytogenes*-specific CD8^+^ T cells in wt and CXCR6^GFP/GFP^ mice at day 5 post secondary infection (data not shown). Finally, it is also possible that the stronger CD4^+^ T cell response of CXCR6^GFP/GFP^ mice might result in enhanced CD4^+^ T cell mediated protection.

In conclusion, our results demonstrate that CXCR6 is neither essential for the generation of T cell responses following *L. monocytogenes* infection nor for the recruitment of activated CD8^+^ T cells to infected tissues. However, CXCR6 appears to regulate the long-term survival and tissue distribution of activated CD8^+^ T cells.

## Supporting Information

Figure S1 **CXCR6 expression on CD8^+^ T cells during** ***L. monocytogenes*** **infection.** CXCR6^+/GFP^ mice were infected with 1×10^4^ *Lm*OVA i.v. and cells from spleens and livers were analyzed at indicated time points. The figure shows the expression of CXCR6 on activated CD44^+^CD62L^−^ CD8^+^ T cells. Symbols give the mean ± SEM, n≥5, and are representative for two experiments.
(TIF)

Figure S2 **Control of** ***L. monocytogenes*** **in CXCR6-deficient mice.** Wt and CXCR6^GFP/GFP^ mice were infected with 5×10^4^ *Lm* i.v. and listeria titers in spleens and livers were determined at indicated time points. Colony forming units (CFU) for individual mice and the median of one experiment are shown, n≥7. Detection limit was 20 CFU. †, one mouse died before determination of titers.
(TIF)

Figure S3 **CXCR6 controls early accumulation of activated CD8^+^ T cells in the liver.** Purified CD8^+^ T cells from wt OT–I and CXCR6^GFP/GFP^ OT–I mice were mixed in a ratio of 1∶1 and a total of 5×10^4^ cells were injected into congenic wt mice i.v. infected with 1×10^5^ *Lm*OVA. Transferred cells from spleens and livers of recipient mice were analyzed at day 3 and day 5 p.i. (A) Percentage of transferred OT–I cells before (dc, donor cells) and three and five days after transfer in spleens and livers of recipient mice. (B) Representative dot plots of CD44 and CD62L expression on transferred OT–I cells. (C) Representative histograms of KLRG1 expression on transferred OT–I cells. Bars give mean ± SEM, n = 3. The experiment was repeated twice with consistent results. MFI, mean fluorescence intensity.
(TIF)

Figure S4 **Distribution, phenotype and cytokine profile of transferred wt and CXCR6^GFP/GFP^ CD8^+^ T cells.** Purified CD8^+^ T cells from wt OT–I and CXCR6^GFP/GFP^ OT–I mice were mixed in a ratio of 1∶1 and a total of 5×10^4^ cells were injected into RAG1^−/−^ mice i.v. infected with 1×10^5^ *Lm*OVA. Transferred cells of recipient mice were analyzed at 5, 10, 15 and 30 weeks p.i. (A) Percentage of transferred OT–I cells in bone marrow, spleens, lungs, blood, lymph nodes and livers on indicated time points. (B) Representative dot plots of CD44 and CD62L expression on transferred OT–I cells in spleens of recipient mice. Numbers show percentages of positive wt (black) and CXCR6^GFP/GFP^ (red) cells. (C) Activation status of transferred OT–I cells in spleens of recipient mice. Bars show distribution of CD44^−^CD62L^+^, CD44^+^CD62L^+^, and CD62L^−^ cells within wt and CXCR6^GFP/GFP^ OT–I cells. (D,E) Cytokine-production of transferred OT–I cells in spleens of recipient mice after *in vitro* stimulation with OVA_257-264_ peptide. (D) Representative histograms of IFN–γ and TNF–α expression of transferred OT–I cells. (E) Percentage of IFN–γ^+^ and TNF–α^+^ transferred OT–I cells. Bars give mean ± SEM of combined results from two independent experiments, n≥6. dc, donor cells.
(TIF)

Figure S5 **Transferred CXCR6^GFP/GFP^ CD8^+^ T cells show similar or reduced expression of exhaustion markers.** Purified CD8^+^ T cells from wt OT–I and CXCR6^GFP/GFP^ OT–I mice were mixed in a ratio of 1∶1 and a total of 5×10^4^ cells were injected into RAG1^−/−^ mice i.v. infected with 1×10^5^ *Lm*OVA. Transferred cells were analyzed at 5, 10, 15 and 30 weeks p.i. For every sample, unstained cells were measured (FMO, fluorescence minus one). (A) Representative histograms of PD–1, LAG3 and CD244 expression on transferred OT–I cells. (B) Difference between MFI (mean fluorescence intensity) of antibody staining and FMO-staining for PD–1, LAG3 and CD244. Bars give mean ± SEM of combined results from two independent experiments, n≥6. dc, donor cells.
(TIF)

Figure S6 **CXCL16 induces survival but no proliferation of CD8^+^ T cells** ***in vitro*** **.** (A) 2×10^5^ cells from wt spleen were stained with the proliferation dye eF670 and incubated for three days with indicated amounts of CXCL16 and IL–15 or anti–CD3 plus anti–CD28 mAbs. For the combination of IL-15 and CXCL16 100 ng/ml of both cytokines were used. Bars give mean MFI (mean fluorescence intensity) ± SEM of eF670 of CD8^+^ T cells. (B) 2×10^5^ cells from wt and CXCR6^GFP/GFP^ spleens were mixed and incubated for three days without stimuli (none), with 300 ng/ml CXCL16 or with anti–CD3 plus anti–CD28 mAbs. Bars give normalized ratios of co-cultured wt and CXCR6^GFP/GFP^ CD8^+^ T cells. Bars give combined results from two independent experiments, n = 4.
(TIF)

## References

[1] Pamer EG (2004) Immune responses to Listeria monocytogenes. Nat Rev Immunol 4: 812–823. 15459672 10.1038/nri1461

[2] Gellin BG, Broome C V (1989) Listeriosis. JAMA 261: 1313–1320. 2492614

[3] Kursar M, Jänner N, Pfeffer K, Brinkmann V, Kaufmann SHE, et al (2008) Requirement of secondary lymphoid tissues for the induction of primary and secondary T cell responses against Listeria monocytogenes. Eur J Immunol 38: 127–138. 18050270 10.1002/eji.200737142

[4] Kurachi M, Kurachi J, Suenaga F, Tsukui T, Abe J, et al (2011) Chemokine receptor CXCR3 facilitates CD8(+) T cell differentiation into short-lived effector cells leading to memory degeneration. J Exp Med 208: 1605–1620. 21788406 10.1084/jem.20102101 PMC3149224

[5] Zhou Y, Kurihara T, Ryseck RP, Yang Y, Ryan C, et al (1998) Impaired macrophage function and enhanced T cell-dependent immune response in mice lacking CCR5, the mouse homologue of the major HIV-1 coreceptor. J Immunol 160: 4018–4025. 9558111

[6] Zhong MX, Kuziel WA, Pamer EG, Serbina N V (2004) Chemokine Receptor 5 Is Dispensable for Innate and Adaptive Immune Responses to Listeria monocytogenes Infection. Infect Immun 72: 1057–1064. 14742553 10.1128/IAI.72.2.1057-1064.2004 PMC321636

[7] Loetscher M, Amara a, Oberlin E, Brass N, Legler D, et al (1997) TYMSTR, a putative chemokine receptor selectively expressed in activated T cells, exhibits HIV-1 coreceptor function. Curr Biol 7: 652–660. 9285716 10.1016/s0960-9822(06)00292-2

[8] Liao F, Alkhatib G, Peden KWC, Sharma G, Berger EA, et al (1997) STRL33, A Novel Chemokine Receptor-like Protein Functions as a Fusion Cofactor for Both Macrophage-tropic and T Cell Line-tropic HIV-1. J Exp Med 185: 2015–2023. 9166430 10.1084/jem.185.11.2015 PMC2196334

[9] Deng H, Unutmaz D, Kewalramani VN, Littman DR (1997) Expression cloning of new receptors used by simian and human immunodeficiency viruses. Nature 388: 296–300. 9230441 10.1038/40894

[10] Deng L, Chen N, Li Y, Zheng H, Lei Q (2010) CXCR6/CXCL16 functions as a regulator in metastasis and progression of cancer. Biochim Biophys Acta 1806: 42–49. 20122997 10.1016/j.bbcan.2010.01.004

[11] Matloubian M, David a, Engel S, Ryan JE, Cyster JG (2000) A transmembrane CXC chemokine is a ligand for HIV-coreceptor Bonzo. Nat Immunol 1: 298–304. 11017100 10.1038/79738

[12] Kim CH, Kunkel EJ, Boisvert J, Johnston B, Campbell JJ, et al (2001) Bonzo/CXCR6 expression defines type 1-polarized T-cell subsets with extralymphoid tissue homing potential. J Clin Invest 107: 595–601. 11238560 10.1172/JCI11902 PMC199429

[13] Matsumura S, Wang B, Kawashima N, Braunstein S, Badura M, et al (2008) Radiation-induced CXCL16 release by breast cancer cells attracts effector T cells. J Immunol 181: 3099–3107. 18713980 10.4049/jimmunol.181.5.3099 PMC2587101

[14] Sato T, Thorlacius H, Johnston B, Tracy L, Xiang W, et al (2005) Role for CXCR6 in Recruitment of Activated CD8+ Lymphocytes to Inflamed Liver. J Immunol 174: 277–283. 15611250 10.4049/jimmunol.174.1.277

[15] Wilbanks A, Zondlo SC, Murphy K, Mak S, Soler D, et al (2001) Expression Cloning of the STRL33/BONZO/TYMSTR Ligand Reveals Elements of CC, CXC, and CX3C Chemokines. J Immunol 166: 5145–5154. 11290797 10.4049/jimmunol.166.8.5145

[16] Shimaoka T, Kume N, Minami M, Hayashida K, Kataoka H, et al (2000) Molecular cloning of a novel scavenger receptor for oxidized low density lipoprotein, SR-PSOX, on macrophages. J Biol Chem 275: 40663–40666. 11060282 10.1074/jbc.C000761200

[17] Bazan JF, Bacon KB, Hardiman G, Wang W, Soo K, et al (1997) A new class of membrane-bound chemokine with a CX3C motif. Nature 385: 640–644. 9024663 10.1038/385640a0

[18] La Porta C a M (2012) CXCR6: the role of environment in tumor progression. Challenges for therapy. Stem cell Rev Rep 8: 1282–1285. 10.1007/s12015-012-9383-6 22678828

[19] Sheikine Y, Sirsjö A (2008) CXCL16/SR-PSOX–a friend or a foe in atherosclerosis? Atherosclerosis 197: 487–495. 18191863 10.1016/j.atherosclerosis.2007.11.034

[20] Hogquist K, Jameson S, Heath W (1994) T cell receptor antagonist peptides induce positive selection. Cell 76: 17–27. 8287475 10.1016/0092-8674(94)90169-4

[21] Unutmaz D, Xiang W, Sunshine MJ, Campbell J, Butcher E, et al (2000) The primate lentiviral receptor Bonzo/STRL33 is coordinately regulated with CCR5 and its expression pattern is conserved between human and mouse. J Immunol 165: 3284–3292. 10975845 10.4049/jimmunol.165.6.3284

[22] Foulds KE, Zenewicz L a, Shedlock DJ, Jiang J, Troy AE, et al (2002) Cutting edge: CD4 and CD8 T cells are intrinsically different in their proliferative responses. J Immunol 168: 1528–1532. 11823476 10.4049/jimmunol.168.4.1528

[23] Paust S, Gill HS, Wang B, Flynn MP, Ashley E, et al (2010) Critical role for CXCR6 in NK cell-mediated antigen-specific memory to haptens and viruses. Nat Immunol 11: 1127–1135. 20972432 10.1038/ni.1953 PMC2982944

[24] Bubnic SJ, Nagy A, Keating A (2005) Donor hematopoietic cells from transgenic mice that express GFP are immunogenic in immunocompetent recipients. Hematology 10: 289–295. 10.1080/10245330500093468 16085541

[25] Van der Voort R, Verweij V, de Witte TM, Lasonder E, Adema GJ, et al (2010) An alternatively spliced CXCL16 isoform expressed by dendritic cells is a secreted chemoattractant for CXCR6+ cells. J Leukoc Biol 87: 1029–1039. 20181724 10.1189/jlb.0709482 PMC3210559

[26] Geissmann F, Cameron TO, Sidobre S, Manlongat N, Kronenberg M, et al (2005) Intravascular immune surveillance by CXCR6+ NKT cells patrolling liver sinusoids. PLoS Biol 3: e113. 15799695 10.1371/journal.pbio.0030113 PMC1073691

[27] Kronenberg M (2005) Toward an understanding of NKT cell biology: progress and paradoxes. Annu Rev Immunol 23: 877–900. 15771592 10.1146/annurev.immunol.23.021704.115742

[28] Arrunategui-Correa V, Sil Kim H (2004) The role of CD1d in the immune response against Listeria infection. Cell Immunol 227: 109–120. 15135293 10.1016/j.cellimm.2004.02.003

[29] Szalay G, Ladel CH, Blum C, Brossay L, Kronenberg M, et al (1999) Cutting edge: anti-CD1 monoclonal antibody treatment reverses the production patterns of TGF-beta 2 and Th1 cytokines and ameliorates listeriosis in mice. J Immunol 162: 6955–6958. 10358132

[30] Emoto M, Yoshizawa I, Emoto Y, Miamoto M, Hurwitz R, et al (2006) Rapid development of a gamma interferon-secreting glycolipid/CD1d-specific Valpha14+ NK1.1- T-cell subset after bacterial infection. Infect Immun 74: 5903–5913. 16988270 10.1128/IAI.00311-06 PMC1594920

[31] Bjordahl RL, Gapin L, Marrack P, Refaeli Y (2012) iNKT cells suppress the CD8+ T cell response to a murine Burkitt's-like B cell lymphoma. PLoS One 7: e42635. 22880059 10.1371/journal.pone.0042635 PMC3413636

[32] Goubier A, Vocanson M, Macari C, Poyet G, Herbelin A, et al (2013) Invariant NKT cells suppress CD8(+) T-cell-mediated allergic contact dermatitis independently of regulatory CD4(+) T cells. J Invest Dermatol 133: 980–987. 23190881 10.1038/jid.2012.404

[33] Mattarollo SR, Yong M, Gosmann C, Choyce A, Chan D, et al (2011) NKT cells inhibit antigen-specific effector CD8 T cell induction to skin viral proteins. J Immunol 187: 1601–1608. 21742969 10.4049/jimmunol.1100756 PMC3150369

[34] Riedel J-H, Paust H-J, Turner J-E, Tittel AP, Krebs C, et al (2012) Immature renal dendritic cells recruit regulatory CXCR6(+) invariant natural killer T cells to attenuate crescentic GN. J Am Soc Nephrol 23: 1987–2000. 23138484 10.1681/ASN.2012040394 PMC3507367

[35] Boisvert J, Kunkel EJ, Campbell JJ, Keeffe EB, Butcher EC, et al (2003) Liver-infiltrating lymphocytes in end-stage hepatitis C virus: subsets, activation status, and chemokine receptor phenotypes. J Hepatol 38: 67–75. 12480562 10.1016/s0168-8278(02)00328-8

[36] Kim CH, Rott L, Kunkel EJ, Genovese MC, Andrew DP, et al (2001) Rules of chemokine receptor association with T cell polarization in vivo. J Clin Invest 108: 1331–1339. 11696578 10.1172/JCI13543 PMC209443

[37] Shields PL, Morland CM, Salmon M, Qin S, Hubscher SG, et al (1999) Chemokine and chemokine receptor interactions provide a mechanism for selective T cell recruitment to specific liver compartments within hepatitis C-infected liver. J Immunol 163: 6236–6243. 10570316

[38] Cose S, Brammer C, Khanna KM, Masopust D, Lefrancois L (2006) Evidence that a significant number of naive T cells enter non-lymphoid organs as part of a normal migratory pathway. Eur J Immunol 36: 1423–1433. 16708400 10.1002/eji.200535539

[39] Shi C, Velázquez P, Hohl TM, Leiner I, Dustin ML, et al (2010) Monocyte trafficking to hepatic sites of bacterial infection is chemokine independent and directed by focal intercellular adhesion molecule-1 expression. J Immunol 184: 6266–6274. 20435926 10.4049/jimmunol.0904160 PMC2921650

