# Supplementary figures and images for: The Function of the Chemokine Receptor CXCR6 in the T Cell Response of Mice against Listeria monocytogenes
Source: PLoS One. 2014 May 15;9(5):e97701. doi: 10.1371/journal.pone.0097701 (PMC4022635; doi:10.1371/journal.pone.0097701)

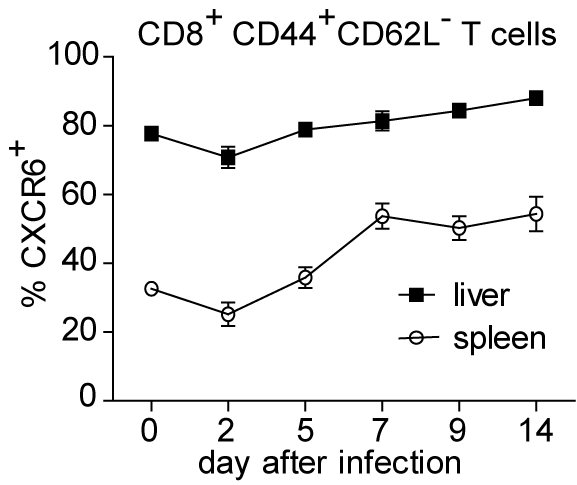

Supplement: Figure S1 — CXCR6 expression on CD8+ T cells during L. monocytogenes infection. CXCR6+/GFP mice were infected with 1×10^4^ LmOVA i.v. and cells from spleens and livers were analyzed at indicated time points. The figure shows the expression of CXCR6 on activated CD44+CD62L− CD8+ T cells. Symbols give the mean ± SEM, n≥5, and are representative for two experiments. (TIF) [file pone.0097701.s001.tif]

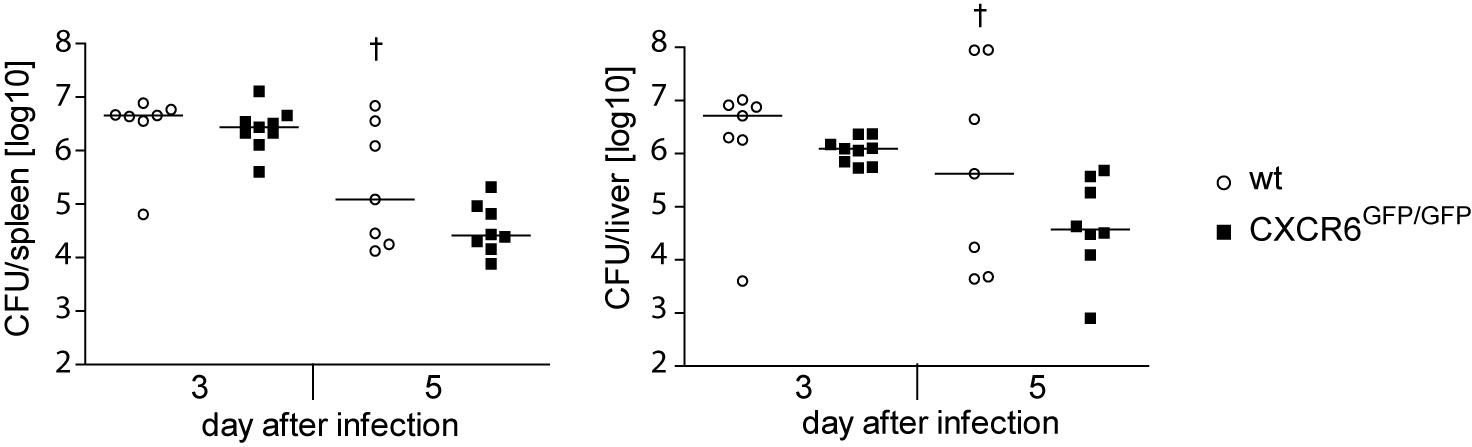

Supplement: Figure S2 — Control of L. monocytogenes in CXCR6-deficient mice. Wt and CXCR6GFP/GFP mice were infected with 5×10^4^ Lm i.v. and listeria titers in spleens and livers were determined at indicated time points. Colony forming units (CFU) for individual mice and the median of one experiment are shown, n≥7. Detection limit was 20 CFU. †, one mouse died before determination of titers. (TIF) [file pone.0097701.s002.tif]

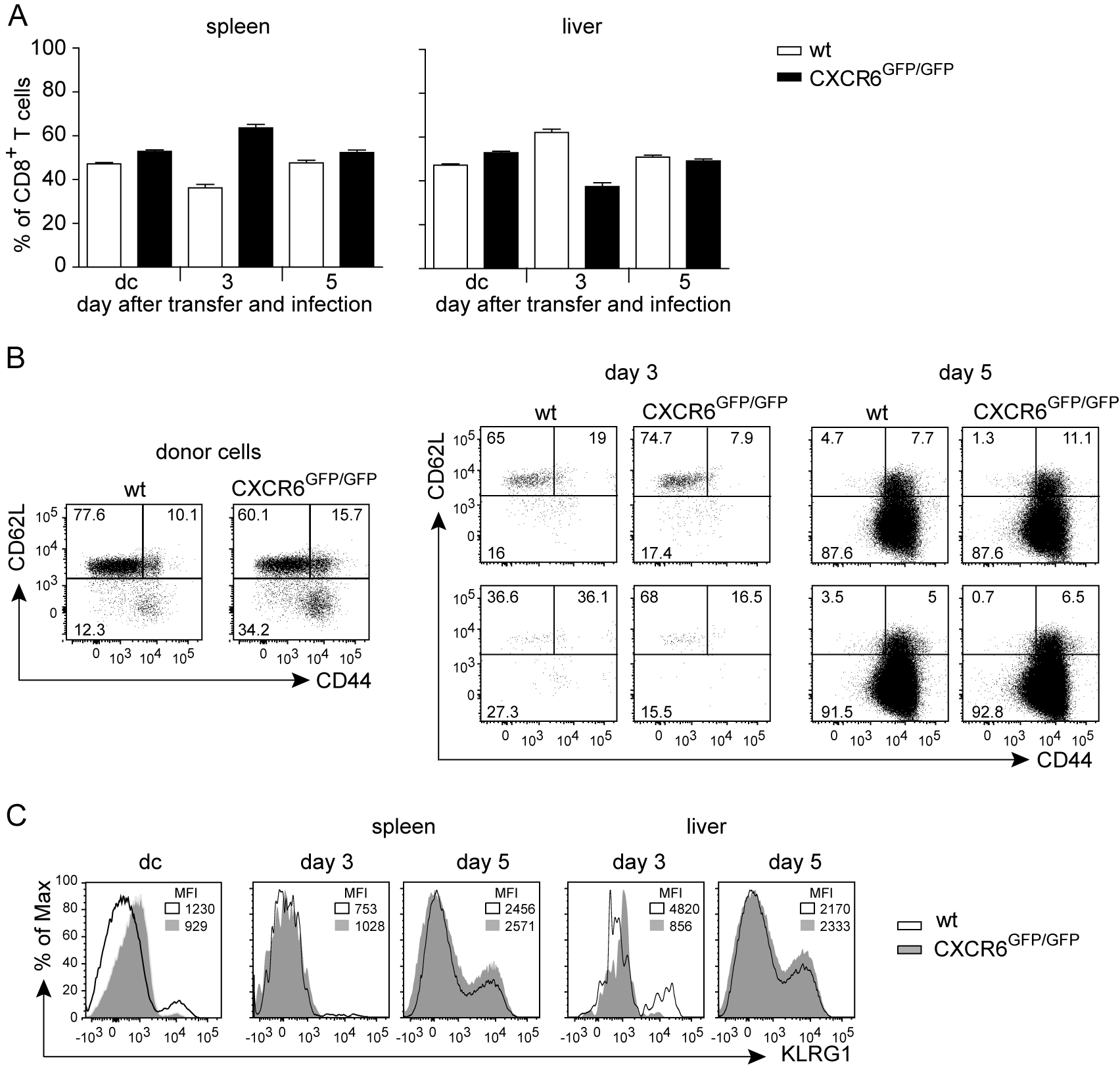

Supplement: Figure S3 — CXCR6 controls early accumulation of activated CD8+ T cells in the liver. Purified CD8+ T cells from wt OT–I and CXCR6GFP/GFP OT–I mice were mixed in a ratio of 1∶1 and a total of 5×10^4^ cells were injected into congenic wt mice i.v. infected with 1×10^5^ LmOVA. Transferred cells from spleens and livers of recipient mice were analyzed at day 3 and day 5 p.i. (A) Percentage of transferred OT–I cells before (dc, donor cells) and three and five days after transfer in spleens and livers of recipient mice. (B) Representative dot plots of CD44 and CD62L expression on transferred OT–I cells. (C) Representative histograms of KLRG1 expression on transferred OT–I cells. Bars give mean ± SEM, n = 3. The experiment was repeated twice with consistent results. MFI, mean fluorescence intensity. (TIF) [file pone.0097701.s003.tif]

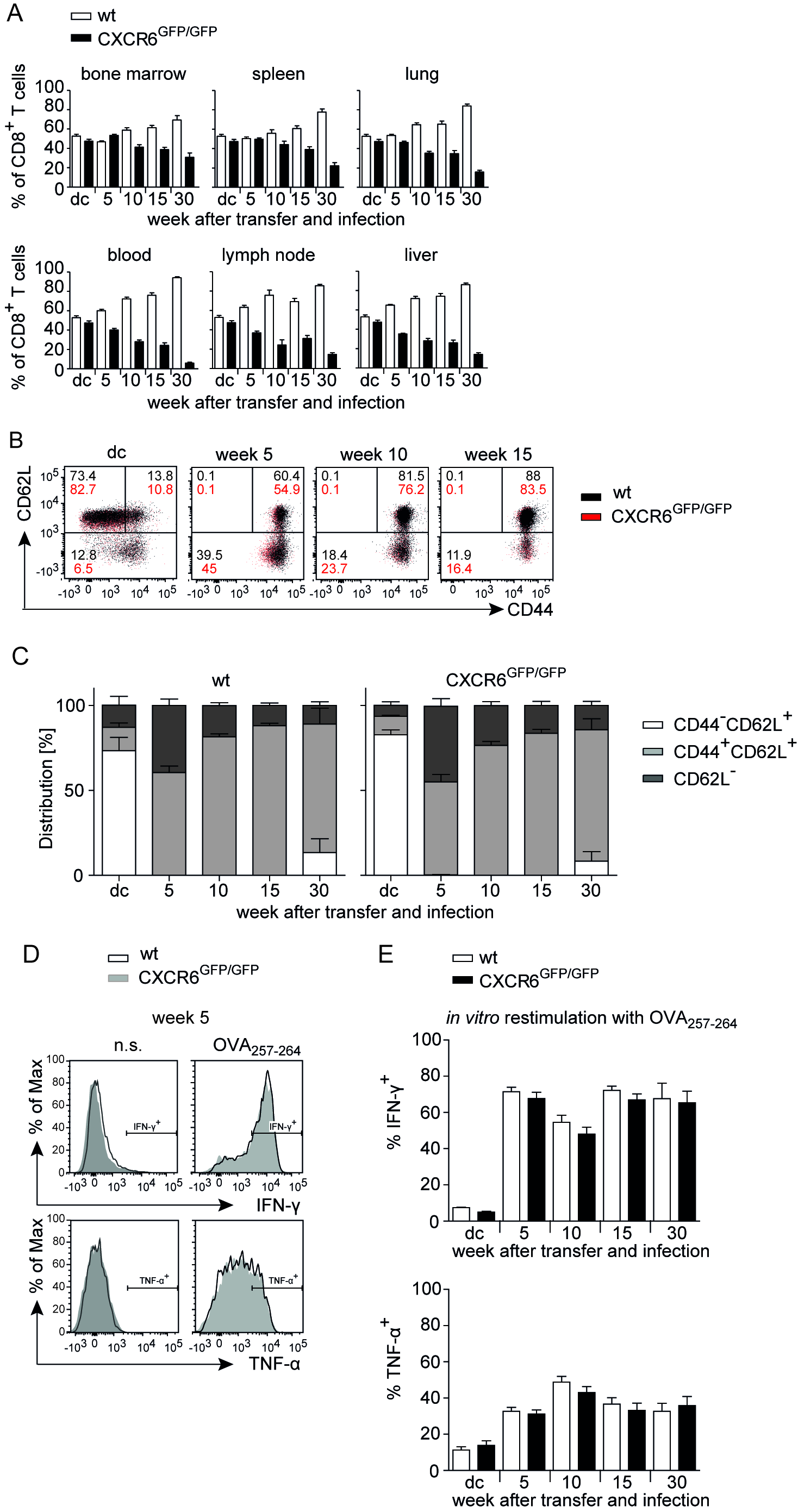

Supplement: Figure S4 — Distribution, phenotype and cytokine profile of transferred wt and CXCR6GFP/GFP CD8+ T cells. Purified CD8+ T cells from wt OT–I and CXCR6GFP/GFP OT–I mice were mixed in a ratio of 1∶1 and a total of 5×10^4^ cells were injected into RAG1−/− mice i.v. infected with 1×10^5^ LmOVA. Transferred cells of recipient mice were analyzed at 5, 10, 15 and 30 weeks p.i. (A) Percentage of transferred OT–I cells in bone marrow, spleens, lungs, blood, lymph nodes and livers on indicated time points. (B) Representative dot plots of CD44 and CD62L expression on transferred OT–I cells in spleens of recipient mice. Numbers show percentages of positive wt (black) and CXCR6GFP/GFP (red) cells. (C) Activation status of transferred OT–I cells in spleens of recipient mice. Bars show distribution of CD44−CD62L+, CD44+CD62L+, and CD62L− cells within wt and CXCR6GFP/GFP OT–I cells. (D,E) Cytokine-production of transferred OT–I cells in spleens of recipient mice after in vitro stimulation with OVA257-264 peptide. (D) Representative histograms of IFN–γ and TNF–α expression of transferred OT–I cells. (E) Percentage of IFN–γ+ and TNF–α+ transferred OT–I cells. Bars give mean ± SEM of combined results from two independent experiments, n≥6. dc, donor cells. (TIF) [file pone.0097701.s004.tif]

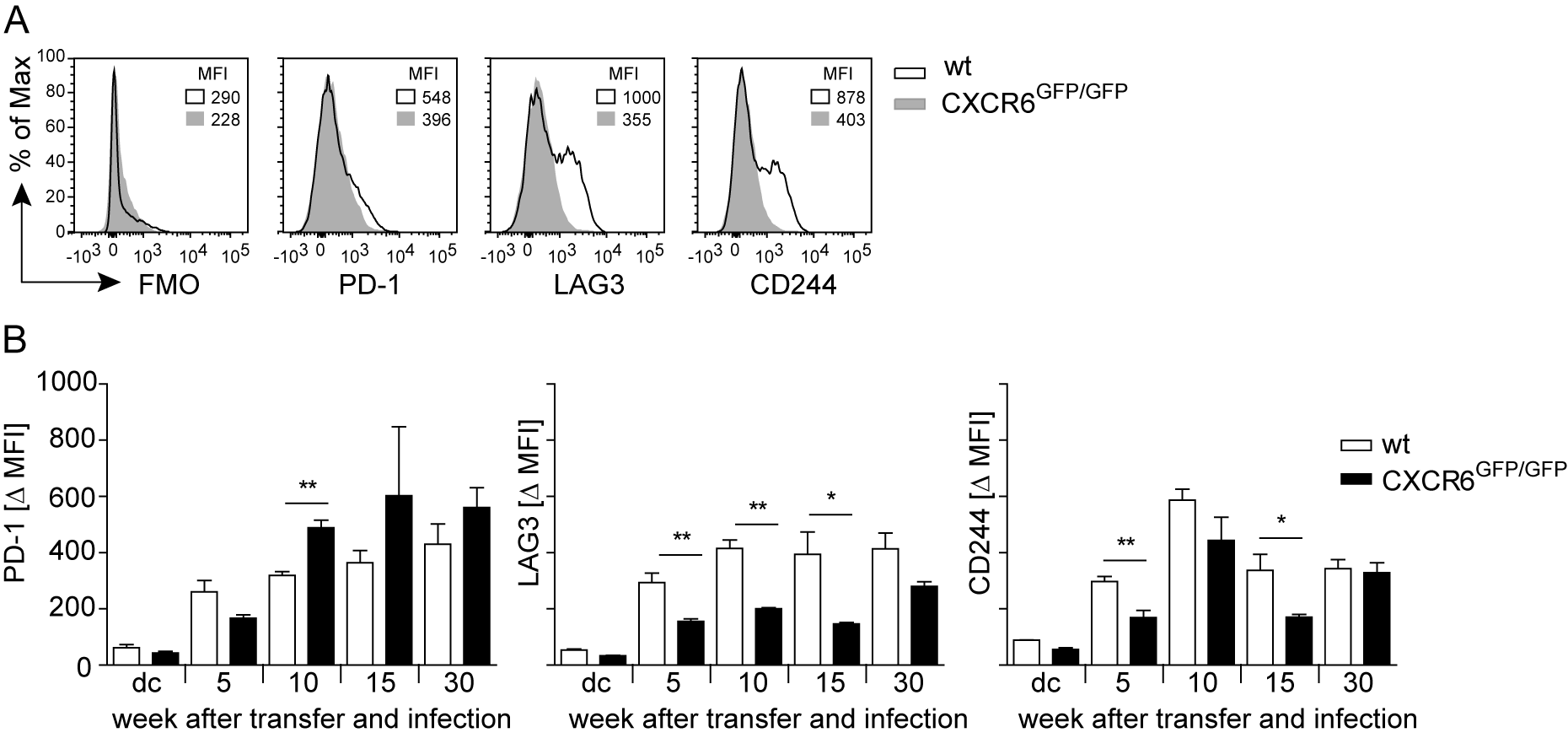

Supplement: Figure S5 — Transferred CXCR6GFP/GFP CD8+ T cells show similar or reduced expression of exhaustion markers. Purified CD8+ T cells from wt OT–I and CXCR6GFP/GFP OT–I mice were mixed in a ratio of 1∶1 and a total of 5×10^4^ cells were injected into RAG1−/− mice i.v. infected with 1×10^5^ LmOVA. Transferred cells were analyzed at 5, 10, 15 and 30 weeks p.i. For every sample, unstained cells were measured (FMO, fluorescence minus one). (A) Representative histograms of PD–1, LAG3 and CD244 expression on transferred OT–I cells. (B) Difference between MFI (mean fluorescence intensity) of antibody staining and FMO-staining for PD–1, LAG3 and CD244. Bars give mean ± SEM of combined results from two independent experiments, n≥6. dc, donor cells. (TIF) [file pone.0097701.s005.tif]

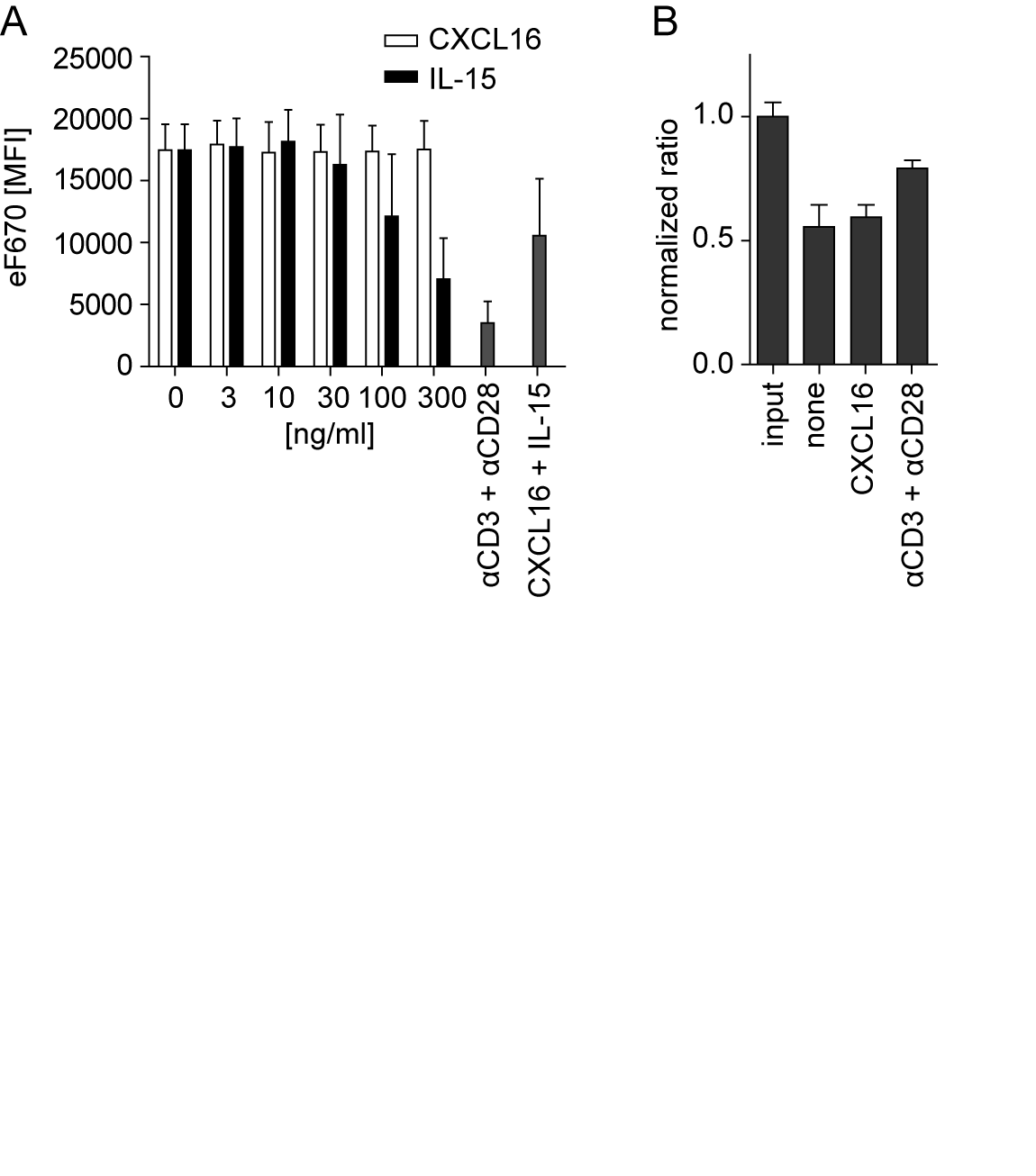

Supplement: Figure S6 — CXCL16 induces survival but no proliferation of CD8+ T cells in vitro . (A) 2×10^5^ cells from wt spleen were stained with the proliferation dye eF670 and incubated for three days with indicated amounts of CXCL16 and IL–15 or anti–CD3 plus anti–CD28 mAbs. For the combination of IL-15 and CXCL16 100 ng/ml of both cytokines were used. Bars give mean MFI (mean fluorescence intensity) ± SEM of eF670 of CD8+ T cells. (B) 2×10^5^ cells from wt and CXCR6GFP/GFP spleens were mixed and incubated for three days without stimuli (none), with 300 ng/ml CXCL16 or with anti–CD3 plus anti–CD28 mAbs. Bars give normalized ratios of co-cultured wt and CXCR6GFP/GFP CD8+ T cells. Bars give combined results from two independent experiments, n = 4. (TIF) [file pone.0097701.s006.tif]
